# OX133, a monoclonal antibody recognizing protein-bound N-ethylmaleimide for the identification of reduced disulfide bonds in proteins

**DOI:** 10.1080/19420862.2016.1152443

**Published:** 2016-03-17

**Authors:** Lisa-Marie Holbrook, Lai-Shan Kwong, Clive L. Metcalfe, Emmanuel Fenouillet, Ian M. Jones, A. Neil Barclay

**Affiliations:** a Sir William Dunn School of Pathology, University of Oxford, Oxford, UK; b CNRS, Institut des Sciences Biologiques, Marseille, France; c School of Biological Sciences, University of Reading, UK

**Keywords:** Allosteric, detection, disulfide bond, labile, maleimide, reduction

## Abstract

In vivo, enzymatic reduction of some protein disulfide bonds, allosteric disulfide bonds, provides an important level of structural and functional regulation. The free cysteine residues generated can be labeled by maleimide reagents, including biotin derivatives, allowing the reduced protein to be detected or purified. During the screening of monoclonal antibodies for those specific for the reduced forms of proteins, we isolated OX133, a unique antibody that recognizes polypeptide resident, N-ethylmaleimide (NEM)-modified cysteine residues in a sequence-independent manner. OX133 offers an alternative to biotin-maleimide reagents for labeling reduced/alkylated antigens and capturing reduced/alkylated proteins with the advantage that NEM-modified proteins are more easily detected in mass spectrometry, and may be more easily recovered than is the case following capture with biotin based reagents.

## Introduction

Disulfide bonds are generated during protein transport in the endoplasmic reticulum (ER). They have long been regarded as protein post-translational modifications that are important for maintaining protein structure and function, but that rarely change post-biosynthesis. Increasing evidence suggests, however, that changes in the cellular redox environment are used to modify certain disulfide bonds at the cell surface, unmasking protein function. These disulfide bonds, termed labile or allosteric bonds, are found in a wide variety of proteins, including those in the extracellular environment and at the cell surface,^1-4^ where they have been shown to play a role in the activation of immune cells,^5^ platelets,^6^ virus entry ^7^ and oncogene function.^8^

Cleavage of allosteric disulfide bonds on the cell surface results from the sequestration to the cell surface of members of the protein disulfide isomerase (PDI) family from the ER where they act as reductants, rather than the oxidant and isomerase activity when ER resident. For example, platelet activation, which utilizes allosteric disulfide bonds to initiate thrombus formation, is inhibited by antibodies to PDI,^9,10^ ERp5 ^11^ or ERp57.^12^ Similarly, inhibition of PDI, using antibodies or thiol-blocking agents, prevents reduction of allosteric disulfide bonds of the HIV envelope protein and inhibits entry of the virus into cells. Accordingly, mapping the changes in disulfide bonding patterns in cell surface proteins is crucial to understanding redox-based control of protein function in normal and pathological states. Using a differential labeling approach in which resident free thiols were first alkylated with methyl-PEO_12_–maleimide and newly-labile disulfides labeled with maleimide-PEO_2_-biotin followed by avidin affinity chromatography and mass spectrometry (MS), Metcalfe et al, identified 87 candidate proteins with labile disulfide bonds on the surface of the murine 2B4 T-cell hybridoma line.^13^ A large range of activatory and inhibitory receptors were found among those proteins containing redox-labile/redox isomerase sensitive disulfide bonds, including integrin adhesion receptors α6, αL, αV, β1, β2 and β3 subunits, T-cell receptor chains, cytokine receptors and members of the CD2/SLAM family of immune-signaling receptors such as CD2, CD150, CD229 and CD244. These data, along with bioinformatics-based analysis of protein structure,^2-4,14^ indicate that labile disulfide bonds are present in many cell-surface proteins and represent an under-investigated area of understanding in the control of cellular function. However, a significant drawback to the identification methods that rely on cysteine labeling and MS is that the modification of cysteines with the large hydrophobic maleimide-biotin moiety results in poor chromatographic, ionisation and fragmentation behavior compared to normally alkylated peptides. A smaller cysteine modification would permit better identification of the peptides containing the modified cysteine residues. Similarly, low pH-mediated separation of antigen and antibody may offer an advantage for downstream procedures compared to a maleimide-PEO_2_-biotin (MPB)-liganded molecule.

We describe the production of a unique monoclonal antibody (mAb), OX133, which recognizes N-ethylmaleimide (NEM) bound to cysteine residues in proteins. OX133 detects NEM-modified proteins on the cell surface, and can be used as an affinity matrix to purify NEM-modified proteins from cell lysates. Crucially, OX133 does not cross react with any other alkylating agent, making it a highly selective reagent for the purification of NEM-labeled proteins and potentially peptides for mass spectrometry-based analysis.

## Results

### Production of mAb that detect NEM-modified proteins

**Figure 1.**
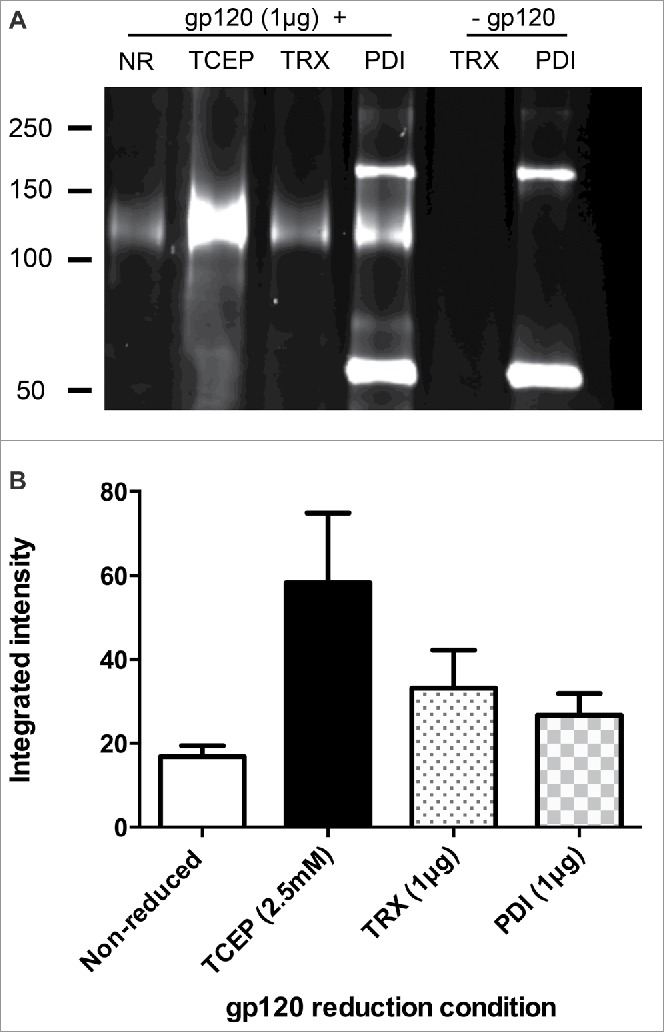
gp120 contains redox labile disulfide bonds which are effectively reduced by TCEP (A) Maltose binding protein-tagged gp120 from HIV-1 CN54 (1 μg - MW 120 kDa) was incubated for 30 min with TCEP (2.5 mM), TRX (1.25 μg) or PDI (1 μg) and alkylated using 5 mM Alexa Fluor 633-conjugated maleimide. Samples were separated by non-reducing SDS-PAGE alongside non-reduced protein and PDI and TRX enzyme only as controls. (B) The integrated intensity of protein bands was quantified using LICOR Odyssey software. Data shown are representative of 3 separate experiments.

HIV-1 surface glycoprotein gp120 contains 9 disulfide bonds that are reported to be labile and susceptible to labeling with NEM following enzymatic or chemical reduction.^15-19^ Complete reduction was determined by testing the reducing abilities of several different reducing conditions followed by labeling the free cysteine residues generated with Alexa Fluor 633-conjugated maleimide and visualization by SDS-PAGE (Fig. 1A). Compared with the biologically relevant enzyme reductants thioredoxin (TRX) and PDI, reduction by tris(2-carboxyethyl)phosphine (TCEP) gave a more reproducible degree of labeling (Fig. 1B), which was complete by 20 minutes of reduction (data not shown). Accordingly, TCEP was used to prepare a batch of reduced gp120 for immunization and, following seroconversion, mAb production was completed using standard procedures. Following the initial cloning, mAbs were screened by ELISA for those that gave strong binding to reduced gp120, but no binding to unreduced gp120. One mAb, termed OX133, was cloned by limiting dilution and analyzed in detail.

### OX133 recognizes NEM-modified cysteines in disulfide bonds in multiple proteins

**Figure 2.**
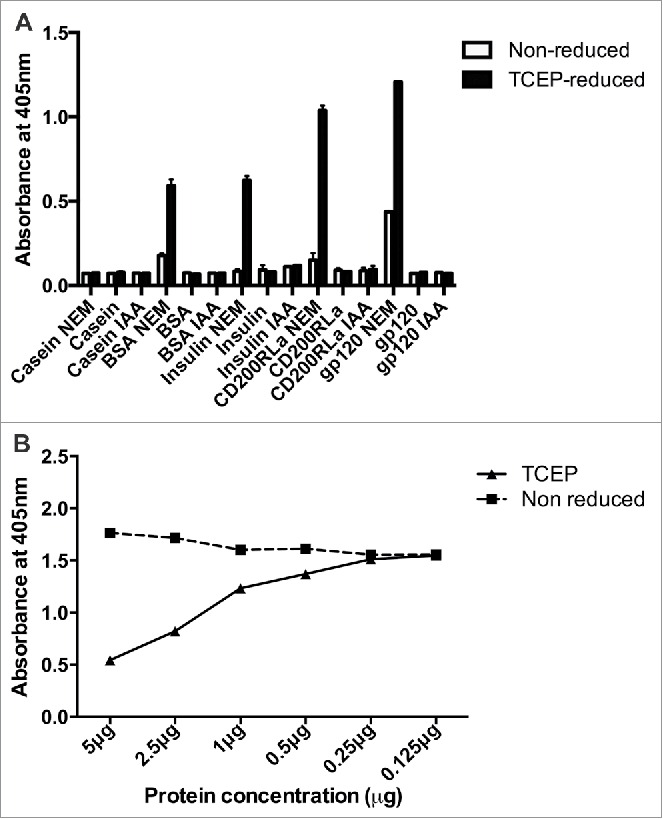
OX133 recognizes reductive changes in multiple proteins containing labile disulfide bonds (A) Target proteins (β-casein, BSA, insulin, CD200RLa or gp120 1 μg/mL) were incubated with PBS, NEM (5 mM) or IAA (5 mM) following reduction with 2.5 mM TCEP for 30 minutes or PBS (non-reduced sample) and coated onto wells of a Maxisorb microtiter plate. Wells were blocked with 0.5% (w/v) protease-free BSA in PBS/Tween for 1 hour at room temperature and alkylated reduction sites detected by incubation with OX133 antibody (0.5 μg/mL in PBS-BSA-Tween) for 1 hour at room temperature. Antibody binding to the plate was determined using anti-mouse alkaline phosphatase conjugate (1:4000) and p-NPP substrate. (B) OX133 specificity for protein-bound NEM was measured by inhibition ELISA. BSA was incubated with either PBS or TCEP (2.5 mM) for 20 minutes, then alkylated with 5 mM NEM for 30 minutes and the unreacted NEM removed. Reduced and alkylated BSA was immobilized to the plate and OX133 binding assessed in competition with free BSA or reduced and alkylated BSA. Antibody binding to the plate was determined using anti-mouse alkaline phosphatase conjugate (1:4000) and p-NPP substrate.

OX133 exhibited the properties expected of a mAb that preferentially recognized reduced gp120, but its specificity for the surrounding peptide sequence was unclear. To assess this, OX133 binding to a variety of non-gp120 proteins that were unreduced or reduced and alkylated with NEM was tested by ELISA. As specificity controls, OX133 failed to react above background levels with TCEP-reduced β-casein, which does not contain any cysteine residues. Similarly, minimal labeling was apparent on a range of proteins, e.g., bovine serum albumin (BSA), insulin, CD200 and gp120, when they were not chemically reduced (Fig. 2A). Upon reduction, however, substantial OX133 mAb binding was observed to all disulfide bond-containing targets with an increase in binding of typically more than 2-fold over the non-reduced form, depending on the number of labile disulfide bonds present. OX133 showed no reactivity to wells containing protein that had not undergone NEM treatment, i.e., proteins reduced but not alkylated, or to each protein treated with the alternative alkylating agent, iodoacetamide (IAA). OX133 is therefore specific for NEM-labeled proteins, and its specificity for NEM in a protein-bound form was further characterized by inhibition ELISA. Capture plates were prepared using BSA reduced with TCEP and alkylated with NEM and OX133 binding titrated in the presence of dilutions of either non-reduced or TCEP-reduced and alkylated BSA in solution (at 5–0.25 μg ml^−1^). As before, OX133 binding was not affected by the presence of non-reduced competitor in the mobile phase, whereas a concentration-dependent competition with the capture target was observed in the presence of free reduced and alkylated protein (Fig. 2B). Free NEM in the mobile phase did not alter binding at any concentration tested (not shown). Thus OX133 is a unique mAb recognizing polypeptide resident, NEM-modified cysteine residues in a sequence-independent manner. OX133 also bound NEM-modified gp120 after tryptic digest (data not shown), suggesting no requirement for full-length protein but no further characterization of any minimal peptide length was completed.

### OX133 recognizes multiple labile disulfide binding sites on the surface of 2B4 cells following reduction

**Figure 3.**
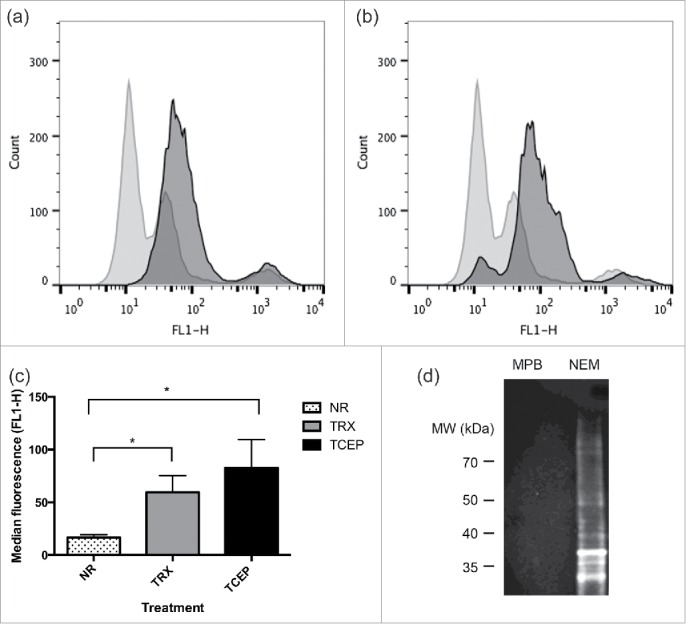
OX133 binds to reduced and labeled proteins at the cell surface and after immobilisation to support surfaces. 2B4 cells (1 × 10^6^) were treated for 30 minutes with TCEP (2.5 mM), TRX (1.25 ug) or PBS as non-reduced (NR) control (both panels). Washed cells were alkylated with NEM (5 mM) and the labile disulfides revealed were labeled with OX133 (1:100) for 20 minutes. Binding was detected by an anti-mouse FITC-conjugate (1:200) followed by flow analysis on a FACSCalibur flow cytometer (BD Biosciences). 10,000 gated (live cell) events were recorded in 4 separate experiments: NR (light gray histogram); TCEP (panel (A)) or TRX (panel (B)) reduction (dark gray histogram). Median fluorescence values for all data revealed that TCEP reduction enhanced NEM labeling of labile disulfides most strongly (*p < 0.05) (C). 2B4 cell lysates from TCEP reduced and NEM alkylated samples were precipitated using sepharose-coupled OX133, separated by SDS-PAGE and Western blotted using OX133 antibody (1:1000) and an anti-mouse conjugate (D).

To determine if OX133 could be used as a tool to recognize NEM labeling of cysteines in their native environment following reduction at the cell surface, the murine 2B4 hybridoma cell line previously shown to have a large number of reducible cell surface resident proteins ^13^ was treated with reducing reagents and alkylated with NEM, followed by OX133 binding and detection by flow cytometry. OX133 mAb labeling increased following treatment with TRX (Fig. 3A - dark gray) or TCEP (Fig. 3B - dark gray) compared to OX133 binding to non-reduced cells (Fig. 3A and B - light gray). The median fluorescent intensity (FL1-H) values for 4 replicates revealed that both TRX and TCEP treatment increased OX133 binding (59 ± 16 and 83 ± 27 respectively) when compared to binding to non-reduced 2B4 cells (17 ± 3) with TCEP reduction revealing more alkylation sites as a result of reducing more labile disulfides (Fig. 3C). OX133 was also coupled to cyanogen bromide-activated sepharose and used to capture NEM labeled proteins from 2B4 cell lysates that had been reduced with TCEP and alkylated with NEM or MPB, as described for Fig. 3A. Precipitated proteins were separated by non-reducing SDS-PAGE and immunoblots were probed using OX133 followed by an anti-mouse conjugate. OX133 detected proteins that had been labeled with NEM, recognizing multiple bands of >100kDa-<30kDa in the reduced and alkylated sample treated with NEM, but not in the sample treated with MPB (Fig. 3D). Thus, the mAb OX133 immunoprecipitates and detects a range of proteins, facilitating the precipitation of specifically NEM-labeled proteins from cell lysates.

## Discussion

We describe the production of OX133, a unique mAb that can selectively detect or purify NEM-labeled cysteine residues in proteins. OX133 was generated by the immunization of mice with reduced and NEM-alkylated gp120 as part of a program to characterize redox intermediates of the gp120 glycoprotein of HIV. HIV gp120 was an ideal immunogen for the generation of reduced disulphide bond discerning antibodies as a result of the 9 disulfide bonds within it structure, 2 of which can be reduced by PDI.^15^ Reduction of gp120 was achieved by reducing agents PDI, thioredoxin and TCEP, but maximal gp120 reduction was observed with TCEP. The mAbs generated were screened for those that distinguished reduced from nonreduced gp120 protein as evidenced by a strongly increased binding to NEM-labeled products. OX133 was found to additionally recognize maleimide labeling of a range of proteins, including BSA, CD200RLa, gp120 and insulin. Conversely, OX133 did not bind to non-labeled proteins or those that were alkylated with iodoacetamide, demonstrating that OX133 specifically recognizes NEM bound to the cysteine residue within a polypeptide. The observed level of OX133 binding broadly correlated with the number of labile disulfides present; BSA contains an undetermined number of labile disulfide bonds,^15^ while 2 of the 3 disulfide bonds in bovine insulin are crucial for maintaining interactions between the A and B chains of the protein and are known to be reduced by thiol isomerase enzymes in vitro.^20,21^ CD200RLa, an activatory paired receptor is formed of 2 Ig-like domains and contains 4 disulfide bonds and 1 unpaired cysteine, explaining a low level of OX133 binding to non-reduced CD200RLa that increased substantially upon TCEP reduction.^22^ OX133 showed minor binding to non-reduced gp120 confirming the finding that 1 free thiol may be present in the mature folded protein.^15^ Generally, background binding to non-reduced samples with no known free Cys was low, but it should be noted that NEM can modify amines at pH >7 .5, and higher backgrounds could result. In addition to solution phase binding, OX133 bound to the murine hybridoma 2B4 cell surface following reduction with either TCEP or thioredoxin and, following coupling to cyanogen bromide-activated sepharose, to reduced and alkylated proteins from these cells.

We conclude that OX133 provides a useful alternative to fluorescently conjugated maleimide compounds for the detection of modified free cysteine residues both at the cell surface and in the study of isolated proteins. Of direct practical value, the purification of NEM-labeled proteins and potentially peptides for the preparation of mass spectrometry samples may prove superior to the use of biotin-maleimide as its use avoids the large biotin moiety and benefits subsequent processing,^13^ although we did not measure directly the relative affinities of NEM modified targets for OX133 verses biotin-maleimide. Additionally, OX133 may prove useful in the study of redox changes associated with pathological conditions as has been the case for the study of protein-bound glutathione in atherosclerotic aortic lesions using a glutathione-dependent antibody.^23^

## Methods and materials

### Materials

Dulbecco's modified Eagle media, protease-free BSA, β-casein, insulin, lentil lectin agarose, alkaline phosphatase-conjugated secondary antibodies, p-nitrophenylphosphate substrate (pNPP), human TRX, rat thioredoxin reductase, cyanogen bromide activated (CNBR) sepharose and NADPH were obtained from Sigma (Poole, UK). Alexa 633-conjugated maleimide was from Invitrogen Life Technologies (Paisley, UK) and TCEP and MPB were from Thermo Fisher Scientific. Purified CD200RLa protein was obtained from Dr Debbie Hatherley (University of Oxford, UK) and purified maltose binding protein (MBP)-gp120 fusion protein was prepared as described.^24^

### Detection of reduced proteins by SDS-PAGE and fluorescent-labeled maleimide

In the initial screening assays, MBP-gp120 was reduced for 30 minutes by incubation with 2.5 mM TCEP, thioredoxin or PDI (both 1 μg). Cysteine residues freed by disulfide reduction were labeled by incubation with 5 mM Alexa 633-conjugated maleimide for 5 minutes. Non-reduced samples were prepared by immediately alkylating protein treated with phosphate-buffered saline (PBS) only. Proteins were separated alongside enzyme-only controls by non-reducing SDS-PAGE and visualized using a LICOR scanner and Odyssey software.

### Monoclonal antibody production and screening

Purified MBP-gp120 was reduced using TCEP (2.5 mM) for 20 minutes and alkylated using NEM (5 mM solubilized in PBS) for 30 minutes. Unbound NEM was removed from protein preparations by buffer exchange into PBS using multiple rounds of dilution and ultrafiltration. Immunizations of Balb/C mice (50 μg/animal) were performed initially in Freund's complete adjuvant with subsequent immunizations using Freund's incomplete adjuvant. MAbs were generated using standard techniques using mouse Sp2/0-Ag14 cells as the hybridoma partner (Bioserv, Sheffield, UK). MAbs recognizing NEM-labeled proteins were detected by ELISA using microtiter plates (Nunc Maxisorp) coated with 100 μL of 1 μg/mL non-reduced or TCEP-reduced gp120 (reduced and alkylated as before) solubilized in 50 mM carbonate-bicarbonate buffer (pH 9.6). Non-specific sites were blocked by incubation with 1% (w/v) protease-free BSA for 1 hour at room temperature and mAbs (100 μL) added for 1 hr with agitation. Antibody binding was detected using alkaline phosphatase-conjugated anti-mouse IgG (1:4000) and p-NPP substrate. MAbs that showed limited reactivity to non-reduced protein and high reactivity to TCEP-reduced protein were selected for cloning and further analysis.

### Detection of NEM-modified proteins by ELISA

Nunc Maxisorp microtiter plates were coated with protein (β-casein, BSA, insulin, CD200RLa or gp120, all at 1 μg/mL, these antigens being non-reduced, TCEP reduced and alkylated with NEM, IAA or not alkylated) solubilized in 50 mM carbonate-bicarbonate buffer (pH 9.6) overnight at 4°C. Wells were blocked by incubation with 1% (w/v) protease-free BSA in PBS/Tween for 1 hour at room temperature and alkylated reduction sites detected through binding of OX133 (0.5 μg/mL in PBS-BSA-Tween) for 1 hour at room temperature and detection.

### Labile disulfide specificity binding ELISA

OX133 specificity was measured by inhibition ELISA: BSA was incubated with either PBS or TCEP (2.5 mM) for 20 minutes then alkylated with 5 mM NEM for 30 minutes and unreacted NEM removed by spin dialysis. Reduced and alkylated BSA and free NEM-treated plates were incubated with OX133 (0.5 μg/mL in PBS-BSA-Tween) for 1 hour at room temperature and detected as before.

### OX133 labeling of 2B4 cells by flow cytometry

The 2B4 murine hybridoma line was cultured in DMEM supplemented with 5% (v/v) fetal bovine serum, 1% (v/v) L-glutamine and 1% (v/v) penicillin-streptomycin and cells grown in an environment containing 5% CO2. Cells (˜10^6^) were harvested at 95%-100% viability and treated for 30 minutes with TCEP (2.5 mM), TRX (1.25 μg) or PBS followed by 2 washes in PBS and alkylation with NEM (5 mM), IAA (5 mM) or MPB (5 mM). Cells were labeled with OX133 (1:100) for 20 minutes and anti-mouse FITC-conjugate (1:200), washed and analyzed on a FACSCalibur flow cytometer (Becton Dickinson). Data for 10,000 events gated on live cells were recorded.

### Preparation of OX133 sepharose and immunoprecipitation

CNBr-activated sepharose was prepared by acidifying in 1 mM HCl. OX133 (1 mg) suspended in coupling buffer (0.1 M NaHCO3, 0.5 M NaCl, pH 8.3) was rotated with prepared CNBr sepharose overnight at 4°C and unbound antibody removed by washing with coupling buffer. Residual active groups on the resin were blocked by incubation with 1 M ethanolamine (pH 9) for 2 hours at room temperature. The resin was washed in the following sequence: TRIS-buffered saline (25 mM TRIS, 140 mM NaCl, pH7.4), 0.1 M glycine (pH 3.5) and PBS. 2B4 cells (1 × 10^6^/mL), reduced and alkylated with either MPB or NEM as described earlier were lysed in PBS containing 1% (v/v) Triton X-100 in the presence of protease inhibitors and 5 mM Iodoacetamide and cell debris removed by centrifugation at 16,000g for 20 minutes. Proteins were then mixed with 100 ul OX133 sepharose and immunoprecipitated overnight at 4°C. Unbound proteins were removed using multiple detergent washes (PBS with 0.1% (v/v) Triton-X100) and precipitated proteins analyzed using SDS-PAGE and immunoblotting.

## References

[1] Hogg PJ. Contribution of allosteric disulfide bonds to regulation of hemostasis. J Thromb Haemost 2009; 7 Suppl 1:13-6; PMID:19630758; http://dx.doi.org/10.1111/j.1538-7836.2009.03364.x 19630758

[2] Schmidt B, Hogg PJ. Search for allosteric disulfide bonds in NMR structures. BMC Struct Biol 2007; 7:49; PMID:17640393; http://dx.doi.org/10.1186/1472-6807-7-49 17640393 PMC1949407

[3] Chen VM, Hogg PJ. Allosteric disulfide bonds in thrombosis and thrombolysis. J Thromb Haemost 2006; 4:2533-41; PMID:17002656; http://dx.doi.org/10.1111/j.1538-7836.2006.02236.x 17002656

[4] Hogg PJ. Disulfide bonds as switches for protein function. Trends Biochem Sci 2003; 28:210-4; PMID:12713905; http://dx.doi.org/10.1016/S0968-0004(03)00057-4 12713905

[5] Metcalfe C, Cresswell P, Barclay AN. Interleukin-2 signalling is modulated by a labile disulfide bond in the CD132 chain of its receptor. Open Biol 2012; 2:110036; PMID:22645657; http://dx.doi.org/10.1098/rsob.110036 22645657 PMC3352089

[6] Essex DW. The role of thiols and disulfides in platelet function. Antioxid Redox Signal 2004; 6:736-46; PMID:15242555; http://dx.doi.org/10.1089/1523086041361622 15242555

[7] Jain S, McGinnes LW, Morrison TG. Thiol/disulfide exchange is required for membrane fusion directed by the Newcastle disease virus fusion protein. J Virol 2007; 81:2328-39; PMID:17151113; http://dx.doi.org/10.1128/JVI.01940-06 17151113 PMC1865930

[8] Cooper CD, Newman JA, Aitkenhead H, Allerston CK, Gileadi O. Structures of the Ets Protein DNA-binding Domains of Transcription Factors Etv1, Etv4, Etv5, and Fev: Determinants of DNA binding and redox regulation by disulfide bond formation. J Biol Chem 2015; 290:13692-709; PMID:25866208; http://dx.doi.org/10.1074/jbc.M115.646737 25866208 PMC4447949

[9] Essex DW, Li M. Protein disulphide isomerase mediates platelet aggregation and secretion. Br J Haematol 1999; 104:448-54; PMID:10086777; http://dx.doi.org/10.1046/j.1365-2141.1999.01197.x 10086777

[10] Cho J, Furie BC, Coughlin SR, Furie B. A critical role for extracellular protein disulfide isomerase during thrombus formation in mice. J Clin Invest 2008; 118:1123-31; PMID:18292814; http://dx.doi.org/10.1172/JCI/10.1016/j.jmb.2010.08.03334134 18292814 PMC2248441

[11] Jordan PA, Stevens JM, Hubbard GP, Barrett NE, Sage T, Authi KS, Gibbins JM. A role for the thiol isomerase protein ERP5 in platelet function. Blood 2005; 105:1500-7; PMID:15466936; http://dx.doi.org/10.1182/blood-2004-02-0608 15466936

[12] Holbrook LM, Sasikumar P, Stanley RG, Simmonds AD, Bicknell AB, Gibbins JM. The platelet-surface thiol isomerase enzyme ERp57 modulates platelet function. J Thrombos Haemost 2012; 10:278-88; PMID:22168334; http://dx.doi.org/10.1111/j.1538-7836.2011.04593.x 22168334 PMC3444690

[13] Metcalfe C, Cresswell P, Ciaccia L, Thomas B, Barclay AN. Labile disulfide bonds are common at the leucocyte cell surface. Open Biol 2011; 1:110010; PMID:22645650; http://dx.doi.org/10.1098/rsob.110010 22645650 PMC3352085

[14] Chiu J, Wong JW, Hogg PJ. Redox regulation of methionine aminopeptidase 2 activity. J Bio Chem 2014; 289:15035-43; PMID:24700462; http://dx.doi.org/10.1074/jbc.M114.554253 24700462 PMC4031554

[15] Kong L, Sheppard NC, Stewart-Jones GB, Robson CL, Chen H, Xu X, Krashias G, Bonomelli C, Scanlan CN, Kwong PD, et al. Expression-system-dependent modulation of HIV-1 envelope glycoprotein antigenicity and immunogenicity. J Mol Biol 2010; 403:131-47; PMID:20800070; http://dx.doi.org/10.1016/j.jmb.2010.08.033 20800070 PMC2950005

[16] Barbouche R, Miquelis R, Jones IM, Fenouillet E. Protein-disulfide isomerase-mediated reduction of two disulfide bonds of HIV envelope glycoprotein 120 occurs post-CXCR4 binding and is required for fusion. J Biol Chem 2003; 278:3131-6; PMID:12218052; http://dx.doi.org/10.1074/jbc.M205467200 12218052

[17] Azimi I, Matthias LJ, Center RJ, Wong JW, Hogg PJ. Disulfide bond that constrains the HIV-1 gp120 V3 domain is cleaved by thioredoxin. J Biol Chem 2010; 285:40072-80; PMID:20943653; http://dx.doi.org/10.1074/jbc.M110.185371 20943653 PMC3000989

[18] Reiser K, Francois KO, Schols D, Bergman T, Jornvall H, Balzarini J, Karlsson A, Lundberg M. Thioredoxin-1 and protein disulfide isomerase catalyze the reduction of similar disulfides in HIV gp120. Int J Biochem Cell Biol 2012; 44:556-62; PMID:22230366; http://dx.doi.org/10.1016/j.biocel.2011.12.015 22230366

[19] Auwerx J, Isacsson O, Soderlund J, Balzarini J, Johansson M, Lundberg M. Human glutaredoxin-1 catalyzes the reduction of HIV-1 gp120 and CD4 disulfides and its inhibition reduces HIV-1 replication. Int J Biochem Cell Biol 2009; 41:1269-75; PMID:19038358; http://dx.doi.org/10.1016/j.biocel.2008.10.031 19038358

[20] Papandreou MJ, Barbouche R, Guieu R, Rivera S, Fantini J, Khrestchatisky M, Jones IM, Fenouillet E. Mapping of domains on HIV envelope protein mediating association with calnexin and protein-disulfide isomerase. J Biol Chem 2010; 285:13788-96; PMID:20202930; http://dx.doi.org/10.1074/jbc.M109.066670 20202930 PMC2859542

[21] Holmgren A. Reduction of disulfides by thioredoxin. Exceptional reactivity of insulin and suggested functions of thioredoxin in mechanism of hormone action. J Biol Chem 1979; 254:9113-9; PMID:39074 39074

[22] Maeda R, Ado K, Takeda N, Taniguchi Y. Promotion of insulin aggregation by protein disulfide isomerase. Biochim Biophys Acta 2007; 1774:1619-27; PMID:17920002; http://dx.doi.org/10.1016/j.bbapap.2007.08.016 17920002

[23] Hatherley D, Lea SM, Johnson S, Barclay AN. Structures of CD200/CD200 receptor family and implications for topology, regulation, and evolution. Structure 2013; 21:820-32; PMID:23602662; http://dx.doi.org/10.1016/j.str.2013.03.008 23602662 PMC3664923

[24] Furuhata A, Honda K, Shibata T, Chikazawa M, Kawai Y, Shibata N, Uchida K. Monoclonal antibody against protein-bound glutathione: use of glutathione conjugate of acrolein-modified proteins as an immunogen. Chem Res Toxicol 2012; 25:1393-401; PMID:22716076; http://dx.doi.org/10.1021/tx300082u 22716076

